# Factors Associated with Poor Eye Drop Administration Technique and the Role of Patient Education among Hong Kong Elderly Population

**DOI:** 10.1155/2019/5962065

**Published:** 2019-03-26

**Authors:** Bonnie Nga Kwan Choy, Ming Ming Zhu, Jason Chun Sum Pang, Jonathan Cheuk Hung Chan, Alex Lap Ki Ng, Michelle Ching Yim Fan, Lawrence Pui Leung Iu, Joseph Shiu Kwong Kwan, Jimmy Shiu Ming Lai, Patrick Ka Chun Chiu

**Affiliations:** ^1^Department of Ophthalmology, LKS Faculty of Medicine, The University of Hong Kong, Hong Kong; ^2^LKS Faculty of Medicine, The University of Hong Kong, Hong Kong; ^3^Department of Ophthalmology, Grantham Hospital, Hong Kong; ^4^Department of Ophthalmology, Prince of Wales Hospital, Hong Kong; ^5^Department of Medicine, LKS Faculty of Medicine, The University of Hong Kong, Hong Kong

## Abstract

**Objectives:**

To identify the risk factors for poor eye drop application technique in treatment-naïve subjects and to assess if patient education can benefit these subjects.

**Methods:**

Chinese subjects above 60 years were recruited. Questionnaires, including Barthel index; Lawton's instrumental activities of daily living (ADL); Fatigue, Resistance, Ambulation, Illnesses, and Loss of Weight (FRAIL) scale; and Montreal Cognitive Assessment (MoCA), were used to correlate with eye drop application technique (before and after patient education) using Spearman correlation analysis. A multiple linear regression was conducted to determine the predictors of successful administration technique and the improvement of technique after education.

**Results:**

The data from 26 subjects (mean age 72) were analyzed. Eye drop instillation technique score improved from 5.42 at baseline to 7.33 after clear instructions. FRAIL score was an independent predictor of baseline score (*p*=0.003), as well as the improvement after patient education (*p*=0.012). Age, sex, education level, visual acuity, Barthel index, MoCA, and ADL score were not correlated with eye drop instillation technique, before nor after patient education.

**Discussion:**

In patients with poor functional status as reflected by FRAIL score, eye drop application is prone to be ineffective. Education with step-by-step instructions could effectively improve the success of eye drop application.

## 1. Introduction

Self-instillation of eye drops is an important technique to acquire for the treatment of various ocular conditions as it is, by far, the most common mode of drug delivery for eye diseases. However, patient's proficiency in self-application of eye drops is not commonly assessed. Previous studies showed that a significant number of patients failed to apply the eye drop properly. One study showed that only 5% of subjects instilled their eye drops properly [1]. Another study of treatment-naïve subjects showed that while 31% could instill one or two eye drops properly, there were also 31% who failed to instill any drop into their eye, and 57% had touched their eyes or ocular adnexa with the bottle tip during instillation [2]. It has been shown that poor eye drop application is related to old age, poor vision, and limited schooling and poor manual dexterity in glaucoma subjects [3]. There was one study which evaluated the demographic and behavioral characteristics that would impact the efficacy of eye drop instillation in glaucoma patients, and it found no association between the Mini-Mental State Examination and successful instillation [4].

For patients with poor eye drop instillation technique, a significant portion of the eye drops cannot be delivered to the ocular surface. This can lead to ineffective treatment response, prematurely running out of medications, and increase medicine wastage. Patients having difficulty in instilling eye drops might be more likely to request more bottles to be prescribed or more likely to return for refill before their appointments. Apart from the extra drug cost, this can increase the likelihood of suboptimal storage of large amount of medications at home and use of expired medications and increases the workload of health-care providers. This is especially problematic in glaucoma patients due to the need to often apply multiple long-term medications. Even if the patient can successfully deliver the eye drop to the ocular surface, poor technique may lead to contamination of the bottle tip, increasing the risk of infection.

Previous studies have used video recording as a mean to assess subjects' eye drop application technique, using various scoring systems [1, 5, 6]. However, most of the current literature studied glaucoma subjects who are already on long-term medications prior to recruitment [4–8]. There are confounding factors on the technique depending on the duration and number of medications and the degree of visual field/visual acuity loss. Furthermore, once the habit of eye drop application technique is established, it may be more difficult to re-educate the patients on the proper technique. Therefore, we want to identify the risk factors for poor eye drop application technique in treatment-naïve subjects and to assess if patient education can benefit these subjects. This helps to correct the improper technique before it becomes a habit. Apart from the general demographic data, we would like to correlate the eye drop administration technique to locally validated questionnaires, including Barthel index; Lawton's instrumental activities of daily living (ADL); Fatigue, Resistance, Ambulation, Illnesses, and Loss of Weight (FRAIL) scale; and Montreal Cognitive Assessment (MoCA), which assess the cognitive and executive functions of our subjects. We plan to identify subjects who are more prone to having difficulties in self-applying eye drops, especially those whose technique do not benefit from patient education. These subjects may require a simplified treatment regime, medical devices which aid with instillation, and assistance from a family member or domestic helper, or should even consider other forms of treatment (for example, laser or surgical intervention for glaucoma and punctal occlusion for dry eyes). If we can identify the risk factors for poor eye drop application, we may be able to develop a simple scoring system for patients requiring long-term eye drops, such as in glaucoma and ocular surface disease, which can alert their ophthalmologist to possible treatment compliance problem and avoid delay in adopting alleviating measures.

We hypothesize that poor cognitive and executive function scores and impaired ADL scores are associated with poor eye drop instillation technique. They are more objectives than demographic data and predict poor self-instillation of eye drop. More education and supervision may be required in such subjects. We also believe eye drop instillation can improve after education. However, education may not benefit those beyond a certain degree of cognitive impairment.

## 2. Materials and Methods

Subjects were recruited from the Ophthalmology clinic of the Queen Mary Hospital and Grantham Hospital, Hong Kong. The study was approved by the Institutional Review Board of the University of Hong Kong/Hospital Authority Hong Kong West Cluster (HKU/HA HKW IRB) and adhered to the tenets of the Declaration of Helsinki. Subjects satisfying the inclusion/exclusion criteria were invited to join the study. The inclusion criteria include subjects aged 60 or above, who are able to give informed consent, can read Chinese, and are native Cantonese speakers. Exclusion criteria include subjects who do not understand written Chinese or spoken Cantonese and those who have been on regular eye drop application in the past 1 year. After explanations about the study, all participating subjects signed an informed consent form.

Demographic data and ophthalmic examination findings were recorded by the investigators, including age, sex, education (level and years), presence of systemic diseases that might possibly interfere with cognitive and executive functions, FRAIL questionnaire which consisted of 5 simple questions to assess the function of the subjects, Chinese versions of Barthel index and Lawton's instrumental ADL to assess subjects' activity of daily living, and Chinese version of MoCA which is a locally validated scoring system to assess the cognitive and executive functions of subjects. Visual acuity (VA) was measured by the Snellen chart first, and then converted into LogMAR value for analysis.

Subsequently, subjects were video-recorded on how they self-instill the eye drops. They were asked to apply a drop of normal saline (0.9%) from a single-use bottle onto the ocular surface of both eyes, without being given prior instructions on how to apply the eye drop. Afterwards, they received education on proper eye drop application technique. After the education session, subjects were reassessed again on their eye drop application technique using video recording. All instructions before and after patient education were delivered by the same research assistant to ensure consistency and comparability of the commands and instructions given. All videos were also recorded by the same research assistant.

The recorded videos were independently graded by 2 investigators who were masked to the subjects' demographic data and the questionnaires results. A score was given to evaluate the effectiveness of eye drop instillation by the following grading system (modified from previously published reports on assessing eye drop application technique [3, 9]):

One point was awarded for each of following action on the checklist, giving a total possible score of 10 points for each eye:

1. Tilting head backward
2. Directing bottle to eye using one hand
3. Pulling down the lower eye lid to expose the inferior forniceal space with fellow hand
4. Able to squeeze the bottle to produce at least one drop
5. Eye drop being successfully applied on the first attempt
6. Less than 3 unsuccessful/missed instillation
7. Only a single drop instilled
8. 2 drops or less used in total
9. Nasolacrimal occlusion after successful instillation
10. Bottle tip remains untouched throughout the recording

The procedure would end after a time limit of 5 minutes for each subject. The scoring would be terminated with no further points awarded after more than 3 unsuccessful instillation attempts or more than 3 eye drops instilled into the eyes.

The average score of both eyes (one score from each masked investigator for the right eye and left eye, respectively) would be calculated, before and after education. The change of the eye drop instillation technique before and after education would be given a score (score after education–score before education).

### 2.1. Data Analysis

The data were presented in the form of number and percentage (%) or mean and standard deviation (SD) and analyzed using Prism GraphPad (version 6, La Jolla, CA) and SPSS software (version 19.0, Armonk, NY). Scores of eye drop instillation before and after patient education were analyzed with paired *t*-test. Spearman correlation analysis and point biserial correlation were conducted to evaluate the correlation between eye drop technique score and other variables, including age, sex, education level, VA, MoCA score, FRAIL score, Barthel index, and Lawton's instrumental ADL score. A multiple linear regression was conducted to determine the predictors of successful administration technique and the improvement of technique after education. Variables enrolled in the regression were those with univariate significance less than 0.2. *p* values less than 0.05 were considered to be statistically significant.

## 3. Results

Twenty-eight subjects were recruited. The videos of 2 of the subjects could not be evaluated and scored properly (the hands blocking part of the view) and were excluded from analysis. Therefore, the data of 26 subjects were available for analysis, including 11 males and 15 females. The mean age was 71.54 years (range: 60–87 years). Five of the subjects had no formal education. Ten subjects received primary education, 10 received secondary education, and 1 had tertiary education. The demographic and clinical cognitive data of all recruited subjects were summarized in Table 1.

**Figure 1:**
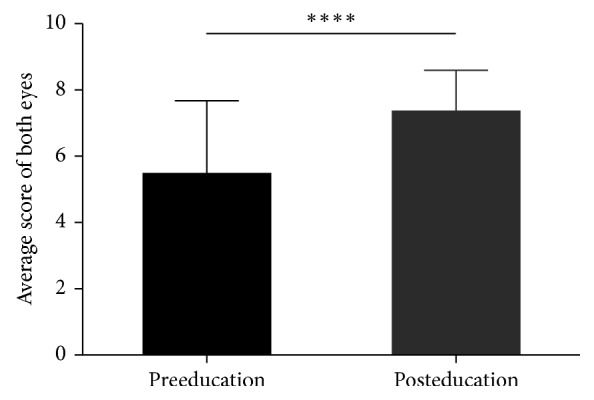
The difference of eye drop technique score between preeducation and posteducation group ^*∗∗∗∗*^; *p* < 0.0001; paired *t*-test.

**Figure 2:**
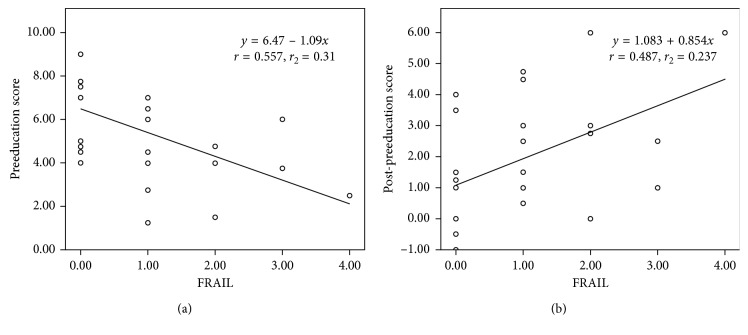
(a) FRAIL score as the predictor of baseline eye drop technique score; (b) FRAIL score as the predictor of the improvement of eye drop technique score after education; multiple linear regression analysis.

The baseline eye drop instillation score before education was 5.42 ± 2.24, and it increased to 7.33 ± 1.27 after education (*p* < 0.0001) (Figure 1). The improvement after education was 1.90 ± 2.01. Univariate analysis showed that sex and FRAIL score were significantly associated with eye drop technique scores (Table 2). Female achieved a lower preeducation score (correlation coefficient = −0.402), but they had more improvement after education (correlation coefficient = 0.392). In multiple linear regression analysis, FRAIL score (*B* = −1.087, *p*=0.003) was the independent predictor for baseline eye drop technique score after adjusting for sex, age, education level, and MoCA score. The regression equation is *y*_1_ = 6.47–1.09*x*_1_, where *y*_1_ is the preeducation score and *x*_1_ is FRAIL score (Figure 2(a)). In addition, FRAIL score (*B* = 0.854, *p*=0.012) was also found to be the independent predictor for the improvement of eye drop technique score after adjusting for sex, age, education level, and MoCA score. The regression equation is *y*_2_ = 1.083 + 0.854*x*_2_, where *y*_2_ is the improvement of education score and *x*_2_ is FRAIL score (Figure 2(b)).

## 4. Discussion

Poor compliance to eye drop prescribed is still one of the major challenges in the management of patients with ocular problems. One of the reasons of nonadherence can be due to poor eye drop instillation technique. Our study was conducted on elderly subjects, with a mean age of 71.54 years, who are usually the age group requiring long-term use of eye drops, mainly lubricants and glaucoma medications. Our study showed that without instruction and education on how to apply the eye drop, the subjects often could not self-apply the eye drop effectively. The average score was only 5.42 points (out of a maximum of 10). None of the subjects could achieve a full score of 10, and one subject scored as low as 1.25. However, after given clear instruction on how to apply the eye drop step by step, the average score improved to 7.33. Two subjects achieved an improvement of 6 points after education. If a score of 7 is considered to be satisfactory eye drop instillation technique, 9 subjects (35%) had satisfactory skill before education, which improved to 17 subjects (65%) after education. Our study is in line with other studies that have shown patients generally do not intrinsically possess good self-application technique for eye drop. However, none of the previous studies assessed the effect of patient education, which our study has demonstrated. Therefore, clear instruction on instillation technique should at least be given to all patients who are prescribed a regular eye drop for the first time, before they take the medication home. In a busy clinic, if one-on-one education may not be practical or feasible, a video or an information leaflet showing step-by-step instructions on how to apply the eye drop should be offered to the patients.

Our study also showed that a higher FRAIL score (which indicates functional impairment) is associated with poor eye drop instillation and is correlated with a worse preeducation score in multivariate analysis. However, those with higher FRAIL score also benefited the most after education. This may be partly because of a lower baseline score to start with. FRAIL is a simple questionnaire consisting of 5 questions. It has been validated in other studies to be an effective tool to assess the functional status of subjects, comparable to other more complicated questionnaires [10, 11, 12]. Apart from the FRAIL score, we did not find any significant correlation of age, education level, and other functional assessment questionnaires. This is in contrast with another study which suggested that poor eye drop application is related to old age, poor vision, limited schooling, and poor manual dexterity in glaucoma subjects. From our study, even elderly subjects with poor vision and limited education are not risk factors for poor eye drop instillation. On the contrary, those with a poor functional status would pose extra difficulty for them to effectively apply the eye drops on their own. However, their technique could be improved when they are provided with clear instruction on how to do it.

This study has some limitations. As a prospective hospital-based self-controlled study, the baseline demographic data and questionnaire scores varied among individuals, which may affect the comparability of the data before and after education. A small sample size in this study might also lead to selection bias. A lager sample size is required in future work to enhance the power of the conclusion.

## 5. Conclusions

To conclude, in patients with poor functional status, eye drop instillation is prone to be ineffective. In those with initial poor technique, education with step-by-step instructions could effectively improve the success of eye drop application, which can reduce bottle contamination and wastage of medication, while maximizing drug effectiveness and compliance.

## Figures and Tables

**Table 1:** Descriptive statistics for demographic and clinical variables.

Characteristics	Value
Mean age, years (SD)	71.54 ± 7.20
Sex, *n* (%)	
Female	15 (57.69%)
Male	11 (42.30%)
Level of education, *n* (%)	
No formal education	5 (19.23%)
Primary	10 (38.46%)
Secondary or higher	11 (42.31%)
Visual acuity (VA), LogMAR^*∗*^ (SD)	
VA of better eye	0.29 ± 0.19
VA of worse eye	0.45 ± 0.29
Weighted average LogMAR VA	0.33 ± 0.20
MoCA score (SD)	23.78 (4.15)
FRAIL score (SD)	0.93 (1.12)
Barthel index (SD)	19.85 (0.53)
Lawton's instrumental ADL score (SD)	7.89 (0.58)

**Table 2:** Univariate analysis for predicting the correlation between eye drop technique scores and demographic and clinical cognitive variables.

	Eye drop application technique score			
	Preeducation		Postpre	
	Correlation coefficient	*p* value	Correlation coefficient	*p* value
Age^#^	−0.33	0.099	0.25	0.217
Sex^*∗*^	−0.40	**0.042**	0.39	**0.048**
Education level^#^	0.33	0.100	−0.23	0.256
Better LogMAR VA^#^	−0.02	0.941	−0.09	0.670
Worse LogMAR VA^#^	−0.13	0.562	−0.09	0.674
Weighted average LogMAR VA^#^	−0.06	0.793	−0.10	0.649
MoCA score^#^	0.36	0.075	−0.27	0.191
FRAIL score^#^	−0.56	**0.003**	0.49	**0.012**
Barthel index^#^	−0.01	0.961	0.02	0.913
Lawton's instrumental ADL score^#^	0.13	0.529	0.19	0.345

^#^Spearman correlation analysis; ^∗^point biserial correlation analysis; variables enrolled were those with univariate significance of ≤0.5.

## Data Availability

The data used to support the findings of this study are available from the corresponding author upon request.

## Abbreviations

ADL: Activities of daily living
MoCA: Montreal Cognitive Assessment
FRAIL: Fatigue, Resistance, Ambulation, Illnesses, and Loss Of Weight
VA: Visual acuity.
